# Direct Priming of CD8^+^ T Cells Persists in the Face of Cowpox Virus Inhibitors of Antigen Presentation

**DOI:** 10.1128/JVI.00186-21

**Published:** 2021-04-26

**Authors:** Leon C. W. Lin, Sarah N. Croft, Nathan P. Croft, Yik Chun Wong, Stewart A. Smith, Swee-Seong Tang, Anthony W. Purcell, David C. Tscharke

**Affiliations:** a John Curtin School of Medical Research, The Australian National University, Canberra, ACT, Australia; b Infection and Immunity Program and Department of Biochemistry and Molecular Biology, Biomedicine Discovery Institute, Monash University, Clayton, VIC, Australia; University of Illinois at Urbana Champaign

**Keywords:** CD8^+^ T cells, T cells, antigen presentation, antigen processing, cowpox virus, immune evasion, vaccinia virus

## Abstract

Vaccinia virus (VACV) was the vaccine used to eradicate smallpox and is being repurposed as a vaccine vector. CD8^+^ T cells are key antiviral mediators but require priming to become effector or memory cells. Priming requires an interaction with dendritic cells that are either infected (direct priming) or have acquired virus proteins but remain uninfected (cross priming). To investigate CD8^+^ T cell priming pathways for VACV, we engineered the virus to express CPXV12 and CPXV203, two inhibitors of antigen presentation encoded by cowpox virus. These intracellular proteins would be expected to block direct but not cross priming. The inhibitors had diverse impacts on the size of anti-VACV CD8^+^ T cell responses across epitopes and by different infection routes in mice, superficially suggesting variable use of direct and cross priming. However, when we then tested a form of antigen that requires direct priming, we found, surprisingly, that CD8^+^ T cell responses were not diminished by coexpression with CPXV12 and CPXV203. We then directly quantified the impact of CPXV12 and CPXV203 on viral antigen presentation using mass spectrometry, which revealed strong but incomplete inhibition of antigen presentation by the CPXV proteins. Therefore, direct priming of CD8^+^ T cells by poxviruses is robust enough to withstand highly potent viral inhibitors of antigen presentation. This is a reminder of the limits of viral immune evasion and shows that viral inhibitors of antigen presentation cannot be assumed to dissect cleanly direct and cross priming of antiviral CD8^+^ T cells.

**IMPORTANCE** CD8^+^ T cells are key to antiviral immunity, so it is important to understand how they are activated. Many viruses have proteins that protect infected cells from T cell attack by interfering with the process that allows virus infection to be recognized by CD8^+^ T cells. It is thought that these proteins would also stop infected cells from activating T cells in the first place. However, we show here that this is not the case for two very powerful inhibitory proteins from cowpox virus. This demonstrates the flexibility and robustness of immune processes that turn on the immune responses required to fight infection.

## Abstract (precis)

CD8^+^ T cells are key to antiviral immunity, so it is important to understand how they are activated. Many viruses have proteins that protect infected cells from T cell attack by interfering with the process that allows virus infection to be recognized by CD8^+^ T cells.

## INTRODUCTION

CD8^+^ T cells are key players in controlling virus infection, but they need to be activated, or primed, in lymph nodes by dendritic cells (DC) before they can use their cytotoxic weapons to kill infected cells. Priming and killing by these cells require an interaction between the T cell receptor of the CD8^+^ T cell with a short virus-derived peptide complexed with major histocompatibility complex class I (MHC-I) on the surface of another cell. For killing, these peptides are processed from the proteins being synthesized in infected cells, which are loaded onto MHC-I in the endoplasmic reticulum (ER) and finally displayed or presented at the cell surface. This is the canonical or “direct” pathway of antigen presentation on MHC-I (1–3). For the priming of CD8^+^ T cells in addition to the direct presentation pathway, DCs can take up viral proteins from their surroundings and process and present peptides from these antigens on MHC-I (2, 4, 5). This second pathway is called “cross” presentation, and the terms direct and cross are also applied to describe the priming of CD8^+^ T cells by these two pathways (2, 6). The way in which CD8^+^ T cells are primed to the plethora of antigens expressed by large viruses, and whether all antigens require the same priming pathway, remain important questions. Cross priming is inevitably required for viruses that cannot infect DCs, but the situation is less clear for viruses that can infect DCs, because both pathways are possible. It has been noted that antigen and, perhaps, peptide characteristics may be factors in the pathway used for priming CD8^+^ T cells (7, 8). Indeed, the antigen requirements that facilitate the most effective CD8^+^ T cell priming by direct and cross presentation have been found to be diametrically opposite by most studies. Direct priming is facilitated when antigens are rapidly degraded, whereas cross priming requires stable protein (9–12).

Due to its frequent use as a model for antiviral immunity and as a vector for vaccines, the priming pathways used by antigens expressed by vaccinia virus (VACV) have received a substantial amount of attention. The balance of evidence points to direct priming being the most frequently used pathway for recombinant and native VACV antigen presentation, noting that there are some exceptions in the literature for particular antigens or recombinant vaccine designs (8, 13). The data supporting direct priming include, first, that VACV-infected dendritic cells have been visualized interacting with CD8^+^ T cells, leading to their activation *in vivo* (14, 15). Second, methods that inhibit cross presentation leave most priming of CD8^+^ T cells by VACV intact (16), with the caveat that these approaches inhibit both pathways to some extent (17). Third, only a VACV engineered to express an MHC-I gene, but not a parental control virus, could prime CD8^+^ T cells to the dominant B8_20_ epitope of VACV in mice with DCs lacking MHC-I (16). Finally, antigens that are unstable and cannot be cross presented are almost always the most immunogenic form of antigen for priming CD8^+^ T cells by VACV (16, 18, 19). The majority of this work done historically used the virulent, Western Reserve (WR) strain of VACV, but more recent work extends these observations to the nonreplicating vaccine strain modified vaccinia Ankara (MVA) (17, 19).

An alternative method to dissect priming pathways for CD8^+^ T cells is to express viral inhibitors of antigen presentation from VACV. These inhibitors are intracellular proteins and would be expected to dampen direct, but not cross, priming. Two groups have tested VACVs that express the human cytomegalovirus proteins US2 and US11, which degrade MHC-I molecules in infected cells. The first found that mice infected with VACVs expressing US2 and US11 primed fewer CD8^+^ T cells that could recognize some, but not all, chromatography fractions of peptides extracted from infected cells (20). The second concurred that US2 and US11 had variable impacts, finding that the route of infection impacted the contribution of presentation pathways (21). These studies suggested that both pathways contribute in an epitope- and infection route-specific manner but have some important caveats. First, degradation of different MHC-I alleles is not the same for US2 and US11; second, US2 can also degrade MHC class II; third, the downregulation of *de novo* viral antigen presentation on infected cells was not especially strong in these studies (20–22). These all suggest that the interpretation of these experiments is not simple.

The discovery of two potent inhibitors of antigen presentation by MHC-I in cowpox virus (CPXV), namely, CPXV12 and CPXV203 (collectively referred to as CPXV12 + 203 here), allowed a similar investigation, albeit for a related orthopoxvirus (23–26). CPXV12 blocks ATP binding to the transporter associated with antigen presentation (TAP), which prevents peptide translocation into the ER (27, 28). CPXV203 retains a broad range of MHC-I allomorphs in the ER (23, 24, 29). These inhibitors have been shown to be potent virulence factors of CPXV via their protection of virus-infected cells from CD8^+^ T cell killing, even though T cell priming is not reduced (30). The interpretation has been that cross priming is sufficient (and necessary) to elicit a maximal CD8^+^ T cell response to CPXV, which is in contrast to VACV, as discussed above. The role of cross priming for CPXV was demonstrated by loss of T cell priming by the virus in *BATF3*^−/−^ (Batf3-knockout [KO]) mice, which lack the major subsets of DCs responsible for cross priming (CD103^+^ and CD8α^+^ DCs) (30, 31). Somewhat counterintuitively, this was shown also for a CPXV lacking the inhibitors of antigen presentation, where there is no *a priori* reason that CPXV-infected DCs might not be able to present antigen. While *BATF3*-dependent DCs are required for cross presentation, there is a lack of evidence that these same DCs also are not required for direct priming, and, indeed, these subsets are in a group that has been noted to induce CD8^+^ T cell priming in general (2, 32). This was addressed in part by one of the CPXV studies noted above by showing that CD8^+^ T cell responses to VACV (as a virus known to require direct presentation, as cited above) were not reduced in Batf3-KO mice (31). However, this finding was not supported by a later publication that found reduced CD8^+^ T cell responses to VACV in Batf3-KO mice (33). This leaves some doubt as to the presentation pathway favored by CPXV and the absolute requirement for cross priming to generate CD8^+^ T cell immunity to avoid inhibition by CPXV12 + 203. Further, studies of CD8^+^ T cell responses are complicated in CPXV infection due to the expression of other viral inhibitors of T cell immunity, including a soluble molecule that blocks costimulation and potentially the induction of interleukin-10 (IL-10) by infection (34–36).

Here, we explore the use of CPXV12 + 203 to dissect direct and cross priming by expressing them from recombinants of the MVA strain of VACV. This revealed that these genes reduce CD8^+^ T cell responses to six of 13 VACV epitopes but, strikingly, not to the dominant B8_20_ epitope, shown by others to require direct priming. We then explored reasons why some epitopes escaped inhibition of priming and found that even some antigens designed to require direct presentation could elicit maximal CD8^+^ T cell responses when coexpressed with CPXV12 + 203. Finally, we used mass spectrometry to measure the extent to which the two CPXV proteins inhibited the presentation of VACV epitopes. This found that presentation was reduced in some cases by 2 orders of magnitude, but this was variable across the peptides, and, despite this inhibition, most remained detectable. Further, in the presence of CPXV12 + 203, B8_20_ was presented at levels higher than those found for most subdominant epitopes in the absence of these inhibitory proteins. Together, these data show that direct presentation can be robust enough to support strong CD8^+^ T cell priming in the face of viral inhibitors of MHC-I. As an inference, cross priming is not necessarily required to explain CD8^+^ T cell responses that are elicited despite viral inhibition of antigen presentation.

## RESULTS

### CPXV12 + 203 expressed from MVA reduce new peptide–MHC-I complexes on the cell surface.

**FIG 1:**
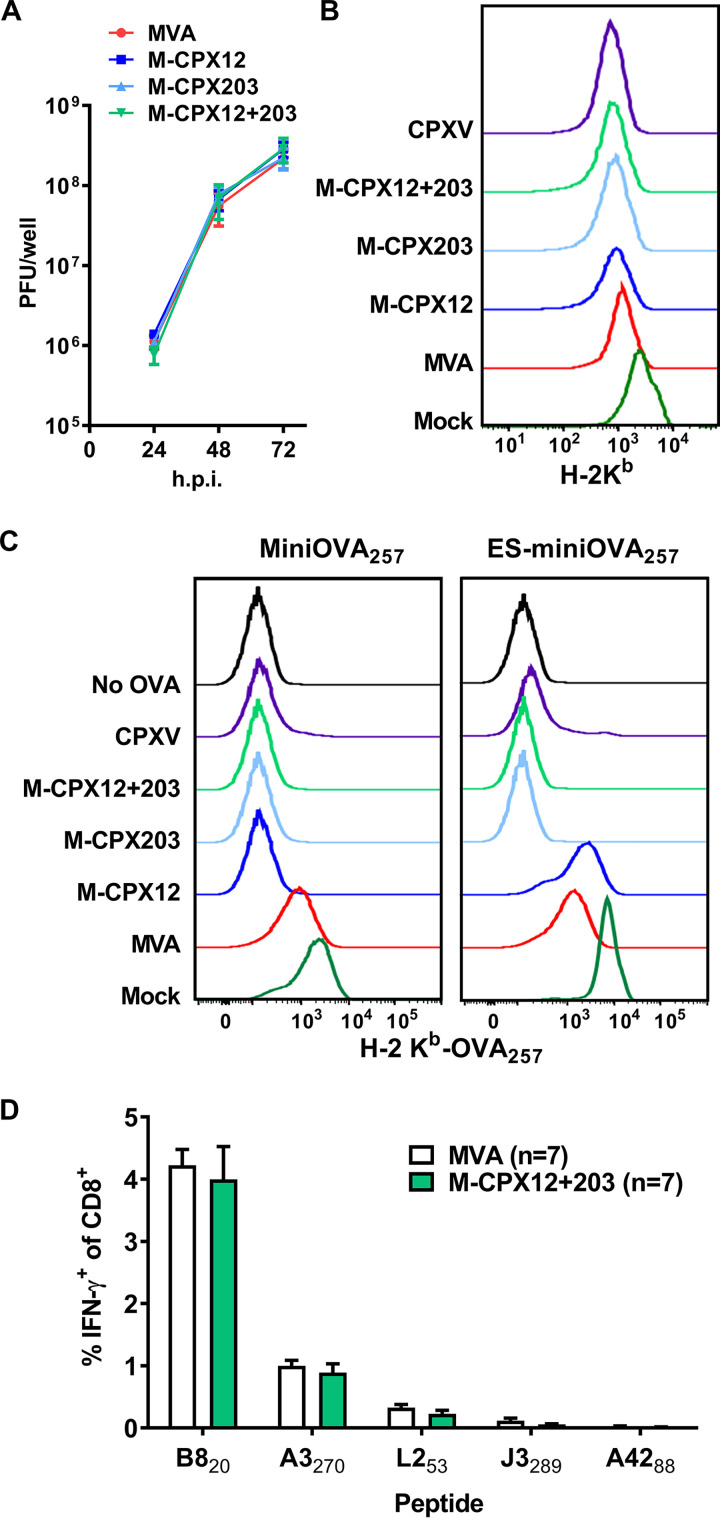
CPXV12 + 203 inhibit direct but not cross presentation. (A) Growth of MVAs expressing CPXV12 and/or CPXV203 as shown after infection of cultures at low multiplicity of infection. Data are the means and ranges of duplicates. (B) 293KbC2 cells were mock infected or infected at an MOI of 5 with the viruses shown, and after 6 h, surface levels of H-2K^b^ were measured by flow cytometry. (C) As per panel B, but coinfections were done with M-MiniOVA_257_ (MiniOVA_257_, left) or M-ESminiOVA_257_ (ES-miniOVA_257_, right) to supply SIINFEKL peptide, and surface H-2K^b^-SIINFEKL was measured. Panels B and C are representative of two independent experiments. (D) Mice were immunized with infected BHK-21 cells (5 PFU/cell for 6 h and then heated at 60°C for 1 h) and CD8^+^ T cell responses measured after 7 days by stimulation of splenocytes with peptides (as shown) and flow cytometry to detect CD8 and intracellular IFN-γ. The percentage of CD8^+^ cells that were IFN-γ^+^ is shown; data are the means and standard errors of the means (SEM) from 7 mice across 3 independent experiments. There were no significant differences for any peptide.

To examine the impact of CPXV12 + 203 on the priming of CD8^+^ T cell responses, we generated three recombinants of VACV strain MVA, one with each gene by itself and one with both CPXV genes. MVA was chosen for this work, because it does not replicate in mouse cells and so cannot replicate and spread in mice, ensuring equal viral loads irrespective of any evasion of immune responses. Further, this strain lacks a homologue of *M2L*, which encodes a protein that interferes with costimulation of T cells and may act to mask the impact of inhibition of antigen presentation. The CPXV genes, including their native promoters, were inserted within the A11R/A12L intergenic region of MVA (37). These viruses, named M-CPX12, M-CPX203, and M-CPX12 + 203, had growth on cells in culture similar to that of their parent, demonstrating that they all had equivalent infectivity (Fig. 1A).

Next, we infected cells with these viruses to determine the effect of CPXV12 + 203 on surface levels of MHC-I. For this, we used 293KbC2 cells, a stable transfectant of 293A cells that expresses mouse H-2K^b^, and noted a small but consistent reduction in K^b^ expression on the cell surface with all the recombinant MVAs at 8 h postinfection (hpi) (Fig. 1B). We reasoned that only a modest difference was seen for both our viruses and for CPXV itself due to the persistence of long-lived peptide–MHC-I complexes that were present before infection. To test the consequence of CPXV12 + 203 on *de novo* antigen presentation, we used coinfection experiments to provide the SIINFEKL peptide of ovalbumin (OVA_257_) from a second recombinant virus and quantified surface K^b^-SIINFEKL complexes by flow cytometry. In our first experiments, coinfections were done with an MVA expressing the minimal OVA_257_ epitope as a minigene (M-miniOVA_257_). On cells infected with any virus expressing one or both CPXV inhibitors, including CPXV itself and our three MVAs, surface K^b^-SIINFEKL complex levels were similar to those on the negative control (Fig. 1C, left). To complement these results and confirm that CPXV12 acts as an inhibitor of TAP, as expected (27, 28), we repeated the experiments described above but supplied SIINFEKL as an ER-targeted minigene (with M-ESminiOVA_257_), such that it bypasses the requirement for TAP. In these experiments, cells that were infected with CPXV, M-CPX203, and M-CPX12 + 203 displayed background levels of K^b^-SIINFEKL, but M-CPX12 was unable to inhibit presentation (Fig. 1C, right). Although we did not verify expression or kinetics, these experiments suggest that CPX12 + 203 are functionally equivalent whether they are expressed from CPXV or from our recombinant MVAs.

Finally, we wanted to show that CPXV12 + 203 do not impede the supply of antigen from infected cells for cross priming by DCs. To do this, we used a well-characterized *in vivo* cross priming experiment in which MHC-mismatched cells were infected with MVA or M-CPX12 + 203 *in vitro* and then heated to inactivate residual virus before being used to immunize mice (17, 19, 38). We then examined CD8^+^ T cell responses to a set of VACV epitopes that can be cross primed in this setting (17, 38) and found that cells infected with either virus elicited a similar-sized response to all specificities (Fig. 1D).

Taken altogether, we concluded that CPXV12 + 203 are very potent inhibitors of MHC-I antigen presentation on infected cells when expressed from our recombinant MVAs, acting as expected and with efficiency similar to that of their native CPXV.

### CPXV12 + 203 reduce CD8^+^ T cell responses to VACV in an epitope- and infection route-dependent manner.

**FIG 2:**
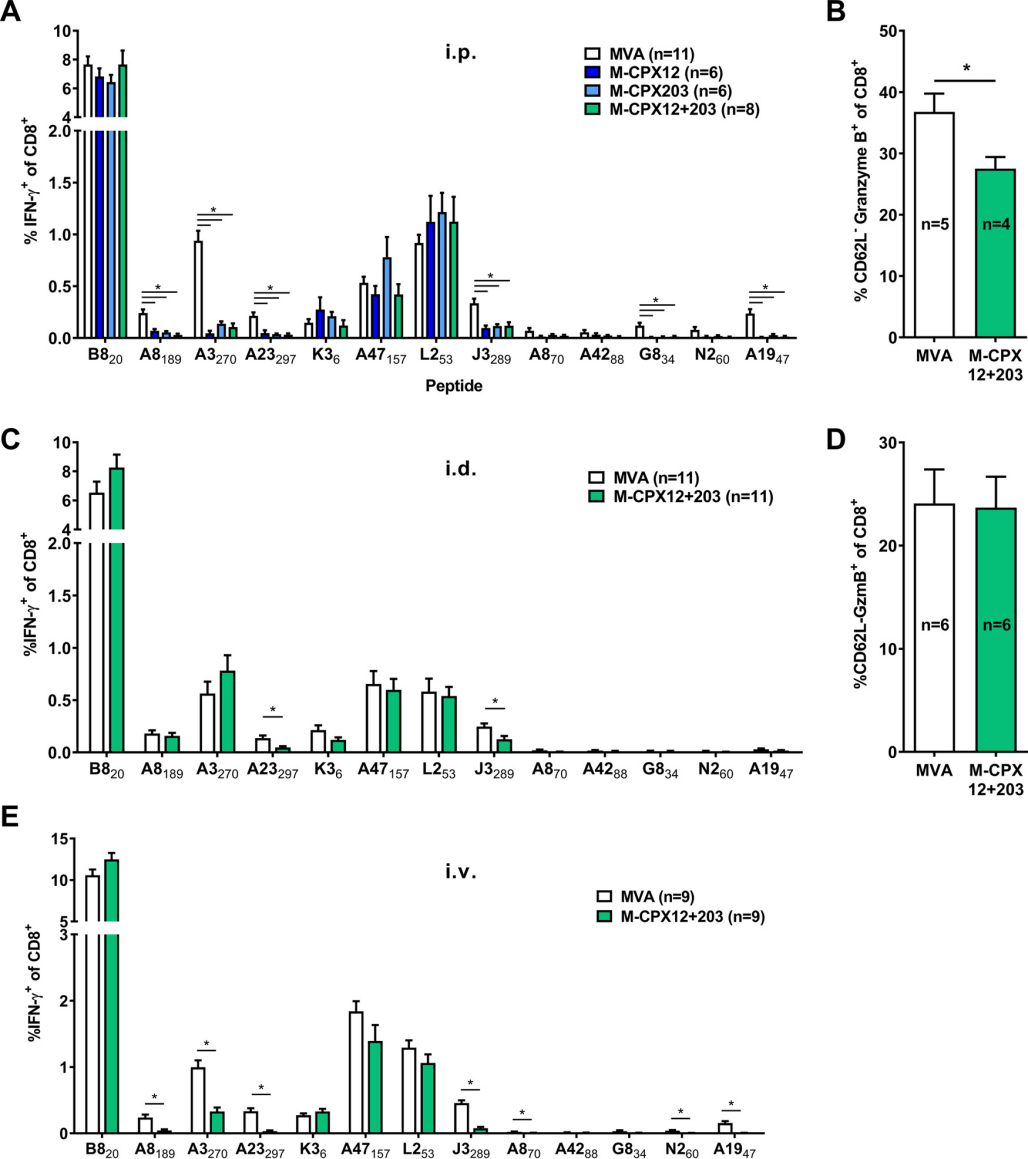
Priming of some, but not all, VACV-specific CD8^+^ responses is reduced by CPXV12 + 203. Mice were infected with the viruses shown by the i.p. (A and B), i.d. (B and C), or i.v. (E) route, and CD8^+^ T cell responses were measured 7 days later. Responses were measured by a short *in vitro* stimulation of splenocytes with peptides (as shown) and flow cytometry to detect surface CD8 and intracellular IFN-γ (A, C, and E) or by enumeration of CD8^+^ cells that were CD62L^−^ and GzmB^+^ by flow cytometry directly *ex vivo* (B and D). Data are means and SEM from the number of mice shown, in each case being pooled from at least 2 independent experiments. Statistical significance was determined by fitting a linear mixed model and ANOVA, using Tukey’s *post hoc* analysis for pairwise comparisons (A and C) or by *t* test (B and D). *, *P* < 0.05; if not marked, not significant.

Next, we assessed the extent to which the CPXV inhibitors of antigen presentation altered the magnitude of VACV-specific CD8^+^ T cell responses primed in mice. First, we examined the responses to a broad range of epitopes at 7 days after intraperitoneal (i.p.) infection with our viruses using a standard assay that comprised a brief *in vitro* stimulation of splenocytes with synthetic peptides followed by intracellular staining for gamma interferon (IFN-γ) (39–43). CD8^+^ T cell responses to some, but not all, peptides were lower in mice infected with the three recombinant MVAs expressing CPXV genes compared with the parent virus (Fig. 2A). These lower responses were significant for A8_189_, A3_270_, A23_297,_ J3_289_, G8_34_, and A19_47_. However, for others, including B8_20_, K3_6_, A47_157_, and L2_53_, all four viruses generated similar CD8^+^ T cell responses. We were somewhat surprised that the results produced by all three recombinant MVAs after infection of mice were indistinguishable, but given this result, the remainder of experiments were done using M-CPX12 + 203 only. Next, we examined the total acute CD8^+^ T cell response to VACV in the spleen 7 days after intraperitoneal infection, using granzyme B (GzmB) and CD62L as markers, as previously established (43, 44). This method found that the fraction of CD8^+^ T cells that were activated by M-CPX12 + 203 was significantly less, being reduced by a quarter compared with MVA (Fig. 2B).

Published experiments with VACVs expressing US11 from cytomegalovirus suggested that inhibition of direct priming reduced CD8^+^ T cell responses after i.p., but not intradermal (i.d.), infection (21). In general agreement with a difference between infection routes, when mice were infected i.d. with MVA or M-CPX12 + 203, there were only two epitopes that primed a significantly lower CD8^+^ T cell response in the presence of the CPXV inhibitors (Fig. 2C). Further, the total size of the activated CD8^+^ T cell response elicited by these two viruses was not significantly different (Fig. 2D). We also explored intravenous (i.v.) infection as a second systemic route, and the results were highly consistent with those seen after i.p. infection (Fig. 2E).

Taken at face value, these data suggest that cross priming can elicit full or partial CD8^+^ T cell responses to many VACV epitopes during systemic infections and is almost entirely sufficient after dermal infection. This conclusion is in keeping with the literature describing experiments with recombinant VACVs expressing US2 and US11 but is at odds with other reports that suggest the primacy of direct presentation for eliciting CD8^+^ T cell immunity to VACV in general.

### Direct presentation can escape inhibition by CPXV12 + 203.

**FIG 3:**
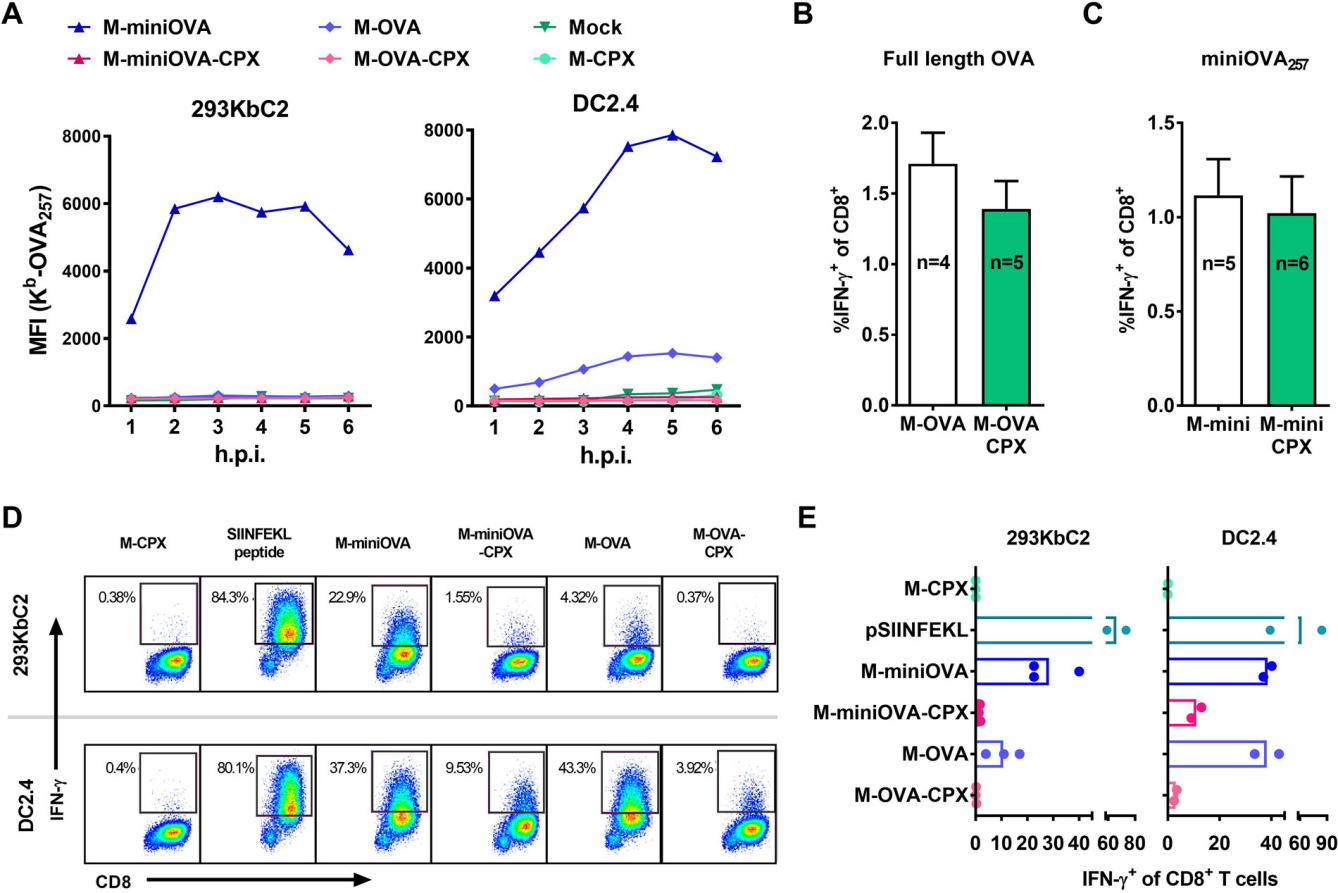
Direct priming despite inhibition by CPXV12 + 203. (A) 293KbC2 or DC2.4 cells were infected with viruses as indicated at 3 PFU/cell, and levels of surface H-2K^b^-SIINFEKL were measured by flow cytometry. Representative data from one of two independent experiments are shown. (B and C) Mice were infected with viruses as shown. CD8^+^ T cell responses to OVA_257_ were measured 7 days later in the spleen. The percentage of CD8^+^ cells that made IFN-γ after a short *in vitro* stimulation with OVA_257_ peptide are shown. Bars show the mean and SEM numbers of mice, which were pooled from two independent experiments. (D and E) Cells were infected as described for panel A or incubated with OVA_257_ peptide for 5 h prior to coincubation with primed OT-I CD8^+^ T cells in the presence of brefeldin A for a further hour, and then surface CD8 and intracellular IFN-γ were measured by flow cytometry. (D) Representative flow cytometry plots from one experiment. (E) Data from 2 or 3 experiments shown with means. Virus names are all abbreviated: CPXV12 + 203, CPX; miniOVA_256_, miniOVA.

An alternative explanation for our data is that CPXV12 + 203 do not completely ablate direct presentation by DCs, and the remaining peptide–MHC-I complexes are adequate to prime a CD8^+^ T cell response. To address this possibility, we engineered our M-CPX12 + 203 virus to express either a minigene version of the SIINFEKL epitope of ovalbumin (miniOVA_257_) or full-length ovalbumin (OVA). MiniOVA_257_ is a 9-amino-acid (aa) peptide with only an initiating methionine in addition to the minimal epitope and, like the vast majority of epitope minigene constructs, cannot be cross primed (10, 12, 19). This is in contrast to full-length OVA protein, which has long been used as a model antigen in cross priming studies (10, 45, 46). Genes for these two forms of OVA were inserted into the thymidine kinase locus of M-CPX12 + 203, such that the new viruses matched a set of previously published MVAs with OVA/miniOVA_257_ (but without CPXV genes) (19). First, we tested the effect of CPXV12 + 203 on antigen presentation *in vitro* by infecting cells and quantifying levels of K^b^-SIINFEKL complexes by flow cytometry. Similar to our experiments described above (Fig. 1C), surface levels of K^b^-SIINFEKL were at background levels on cells infected with MVAs that expressed CPXV12 + 203 irrespective of the form of OVA being expressed (Fig. 3A). This was the same in 293KbC2 and DC2.4 cells; however, only the latter were able to process and present detectable amounts of OVA_257_ from full-length OVA protein.

We then infected mice with these viruses to ask whether CPXV12 + 203 would impede CD8^+^ T cell responses to OVA_257_ when this antigen is expressed in a form that requires direct priming (miniOVA_257_) compared with a form that can be cross primed (OVA). CPXV12 + 203 did not alter CD8^+^ T cell responses generated by full-length OVA, which was not unexpected, because this form of antigen can use cross presentation to avoid interference by the CPXV inhibitors (Fig. 3B). Surprisingly, however, the same result was observed when the source of antigen was miniOVA_257_, which requires direct priming (Fig. 3C). These data suggest that at least for one antigen, the CPXV proteins are not efficient enough to reduce direct presentation *in vivo* to a level that impedes priming of CD8^+^ T cell responses.

To explore the possibility that some direct antigen presentation can escape inhibition by the CPXV proteins, we returned to an *in vitro* setting, but this time using OT-I CD8^+^ T cells to detect presentation instead of direct antibody labeling to improve sensitivity. In this experiment, we tested the ability of infected cells to restimulate *in vitro*-primed OT-I CD8^+^ T cells such that they make IFN-γ. Cells infected with either version of OVA in the absence of the CPXV inhibitors were able to stimulate IFN-γ production by the OT-I cells, with DC2.4 being more efficient than 293KbC2 (Fig. 3D and E). For viruses coexpressing CPXV12 + 203, stimulation was significantly reduced but was above background for miniOVA_257_ in 293KbC2 and for both forms of OVA in DC2.4. These findings show that direct presentation is not ablated by CPXV12 + 203 and that this most obviously was the case on a DC-like cell line.

### CPXV12 + 203 reduce but do not ablate direct presentation of most VACV epitopes.

**FIG 4:**
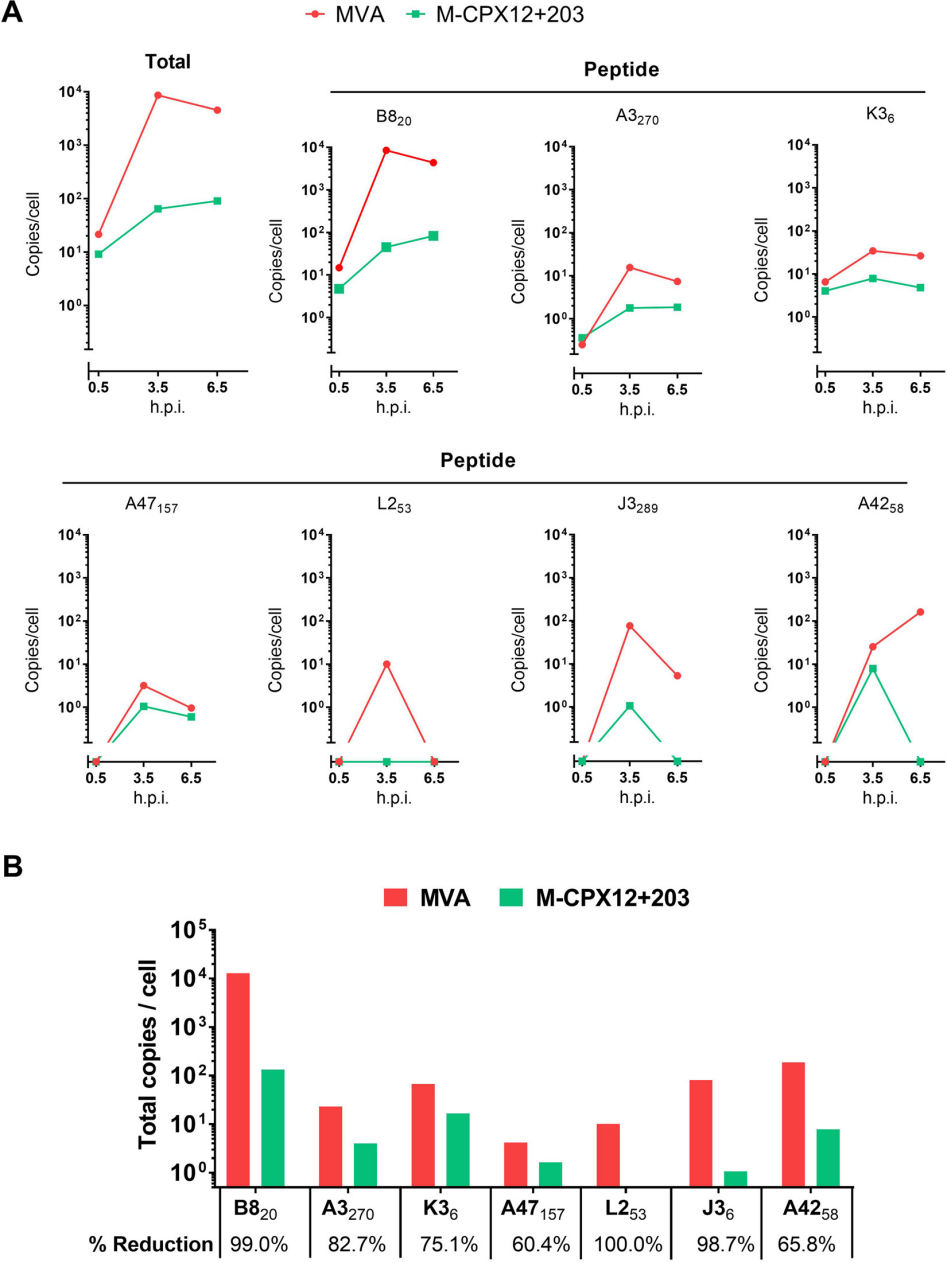
Inhibition of antigen presentation by CPXV12 + 203 is variable across epitopes and typically incomplete. DC2.4 cells were infected with MVA or M-CPX12 + 203 at 3 PFU/cell for 0.5, 3.5, and 6.5 h, and quantification of peptides present on H-2K^b^ and H-2D^b^ was done by LC-MS/MS using a multiple reaction monitoring (MRM) protocol. (A) Kinetics of epitope presentation for either the total or individual peptides as shown. (B) Presentation of epitopes was summed across the 3 time points to give a single value for each virus, and the percent reduction of each on cells infected with M-CPXV12 + 203 compared with MVA is shown under the graph.

Having shown that direct presentation of a model antigen can persist despite inhibition by CPXV12 + 203, we wanted to extend this to a broader range of epitopes. To do this, we used mass spectrometry to quantify the presentation of a set of VACV epitopes on DC2.4 cells infected with MVA and M-CPX12 + 203. The presentation of all epitopes was reduced in the presence of the CPXV proteins, but with the exception of L2_53_, all could be detected at one or more time points after infection (Fig. 4A). Looking at the kinetics of presentation, for those epitopes detectable already 30 min after infection, there was relatively little reduction in abundance in the presence of the CPXV inhibitors. In contrast, for two epitopes, presentation was completely abolished by CPXV12 + 203 at the latest time. When we combined presentation at all times to estimate the total reduction due to CPXV12 + 203 for each epitope, a surprising amount of variation was observed across the peptides (Fig. 4B). This level of reduction was not obviously related to the amount of presentation from wild-type MVA, the kinetic class of the protein, or the affinity of the peptide for MHC-I (43). Two other observations stand out. First, this experiment showed the striking dominance of B8_20_ in presentation. Indeed, despite this epitope being one of the most severely affected by CPXV12 + 203 (99% reduction), it still achieved a higher abundance on cells infected with M-CPX12 + 203 than most other epitopes we measured reached after infection with MVA. Second, L2_53_ is the only epitope that is completely undetectable in the presence of the CPXV proteins. Interestingly, this epitope is poorly presented even on cells infected with wild-type MVA, which contrasts with its relatively strong immunogenicity *in vivo*.

We conclude from these mass spectrometry data that even when combined, CPXV12 + 203 strongly inhibit, but generally fail to ablate, the presentation of epitopes on infected DCs and that their influence on presentation varies by epitope.

## DISCUSSION

CPXV12 + 203 have been shown to be highly effective inhibitors of antigen presentation on MHC-I *in vitro*, and this is reflected in the protection of infected cells from CD8^+^ T cell attack *in vivo*. In light of this high efficiency, the finding that these proteins do not inhibit the priming of CD8^+^ T cells *in vivo* has been interpreted as evidence that CPXV priming occurs by cross presentation. Here, we show that when expressed from a recombinant MVA, the CPXV inhibitors can inhibit priming of some epitopes when the virus is given by the i.p. route. Interestingly, this was not the case for the B8_20_ epitope, which was unexpected, because others have shown by multiple methods that this peptide primes CD8^+^ T cells by direct presentation. Probing this further, we found that presentation of VACV antigens by DCs is sufficiently robust that even where there is suppression by CPXV12 + 203, some epitopes still can prime a maximal CD8^+^ T cell response. This apparent incomplete inhibition of antigen presentation by CPXV12 + 203 was then supported by the use of highly sensitive methods *in vitro*. It remains possible that these genes do not behave entirely in the same manner in MVA as in CPXV, potentially due to the action of other VACV genes or the absence of certain CPXV genes. However, the simplest explanation is that CPXV12 + 203, like almost all immune evasion mechanisms, simply fails to be 100% effective, and this would apply equally in CPXV as in MVA.

These results have implications for how we interpret experiments that use inhibitors of antigen presentation to dissect priming pathways for CD8^+^ T cells. First, if inhibition of priming is observed, as was the case for some of the MVA epitopes (e.g., A8_189_, A3_270,_ A23_297_, J3_289_, G8_34_, and A19_47_), the interpretation remains simple. It can be concluded that direct presentation is required to prime at least the portion of the response lost when the inhibitors are present. For any residual response to these epitopes and where priming is unaffected by inhibitors, the interpretation is more complex. It remains possible that cross presentation is sufficient for the priming of these CD8^+^ T cell responses. However, we show here that an alternative is that the inhibitors fail to reduce presentation by infected DCs below the abundance threshold required for maximal priming. The final possibility is that a combination of direct and cross presentation primes these CD8^+^ T cells.

These considerations should be applied to the older literature that used US2 and US11 to dissect priming routes, especially in light of the *in vitro* data in those papers that suggested substantial residual direct presentation despite expression of the inhibitors (20, 21). Having noted this, our results with regard to route support the earlier report by Shen et al. (21), in that we also found that an inhibitor of antigen presentation reduced priming to some epitopes after i.p., but not i.d., infection. This is despite using different strains of VACV. While our findings agree, our interpretation differs, because we are no longer confident that the underlying mechanism is a different requirement for direct and cross priming by these routes. Indeed, it has been shown recently that virulent VACVs and MVAs with antigens optimized for direct priming are typically also the best at inducing CD8^+^ T cell responses after i.d. infection (19). Therefore, the reduced ability of CPXV12 + 203 to block CD8^+^ T cell priming after i.d. infection may be caused by the use of cross priming or the DCs involved are especially efficient at direct priming.

Notwithstanding the caveats noted above, from the totality of the data here, it seems reasonable to comment on the most likely priming pathway for at least two VACV epitopes, namely, B8_20_ and L2_53_. B8_20_ is presented at very high levels after infection of cells and, indeed, is so efficiently presented that it remains at more than 100 copies per cell despite a 99% reduction in the presence of CPXV12 + 203. Given this very high residual abundance in the face of inhibition, it seems likely that very efficient direct presentation explains why CD8^+^ T cell responses to this epitope are unaffected by CPXV12 + 203. L2_53_ provides a contrasting picture. Our mass spectrometry data show that L2_53_ is very poorly presented on infected cells, and this presentation can be reduced below our limit of detection (<1 copy per cell) by CPXV12 + 203. Despite this, L2_53_ is relatively immunogenic, ranking second or third behind B8_20_ in the MVA immunodominance hierarchy, and the priming of this epitope is unaffected by CPX12 + 203 *in vivo*. Together, these findings suggest that L2_53_ elicits CD8^+^ T cells by cross priming, and this is why the CPXV inhibitors fail to reduce L2_53_-specific responses. These two examples support the evidence from others that priming pathway can differ among antigens, epitopes, and constructs for orthopoxviruses, even if direct priming is most consistently observed (8, 13, 47–50). Further, some studies suggest that direct and cross presentation pathways can be utilized sequentially in the priming of epitopes (48, 51). In addition, we have only studied systemic responses (as observed in the spleen), and the rules might differ for particular T cell subsets, for example, tissue resident memory cells (52).

Finally, our results here are a reminder that no matter how strong and absolute viral immune evasion mechanisms can appear, especially in some *in vitro* or biochemical settings, immune processes are highly efficient and are rarely completely inhibited. Indeed, in the case of CPXV, even with these two powerful inhibitors of antigen presentation, the virus has also maintained interference with costimulation as an independent mechanism to inhibit T cell priming (35, 36). It is important to note that CPXV and VACV appear to occupy a broad niche in the wild that most likely includes a range of rodents as well as incidental host species, such as humans and their domestic animals (the bovine host implied in the names of these viruses being a red herring) (53–55). While there is no apparent species specificity in the function of CPXV12 or CPXV203 between humans and mice (23–26), it remains possible that their efficiency in blocking antigen presentation varies across other hosts, and this has not been captured in our experiments here. Nevertheless, we speculate that the incomplete inhibition of antigen presentation we show suggests that CD8^+^ T cells remain a threat to the fitness and continued survival of CPXV and, most likely, orthopoxviruses in general.

## MATERIALS AND METHODS

### Mice.

Specific-pathogen-free, female, C57BL/6 or OT-I transgenic mice, between 7 and 14 weeks old, were obtained from the Australian Phenomics Facility (Canberra, Australia) or the Animal Resources Centre (Perth, Australia). All experiments involving mice were conducted in accordance with relevant ethical requirements, approved by the Australian National University Animal Ethics and Experimentation Committee (protocols F.BMB.38.08, A2011.001, and A2013.037).

### Cells.

All cells were maintained in Dulbecco’s modified Eagle medium (DMEM) supplemented with 10% fetal bovine serum (FBS) and l-glutamine. BHK-21 cells were used to grow and titrate all MVAs and as antigen donor cells used for immunizing mice to examine cross priming. 293KbC2 (56) and DC2.4 (57) cells were used to examine direct antigen presentation *in vitro*.

### Nonrecombinant viruses and virus growth.

All viruses used in this study are listed in Table 1.

**TABLE 1:** Viruses used throughout this study

Name^*a*^	Full name^*b*^	Description^*c*^	Source or reference
CPXV	Cowpox virus (Brighton red)	Cowpox virus strain Brighton red	G. L. Smith
MVA	Modified vaccinia Ankara	Parent MVA (MVA 1974/NIH clone 1; MVA572.FHE-22.02.1974 plaque purified at NIH)	B. Moss
M-CPX12	MVA-A11R/A12L-CPXV12	MVA with *CPXV12* and its native promoter inserted within the noncoding region between *A11R* and *A12L* and transcribed toward the right	This study, 37
M-CPX203	MVA-A11R/A12L-CPXV203	MVA with *CPXV203* and its native promoter inserted within the noncoding region between *A11R* and *A12L* and transcribed toward the left	This study, 37
M-CPX12 + 203	MVA-A11R/A12L-CPXV12 + CPXV203	MVA with *CPXV12* and *CPXV203* with their native promoters inserted within the noncoding region between *A11R* and *A12L* such that they are transcribed toward each other (gene arrangement, left to right, *A11R*, *CPXV12*, *CPXV203*, *A12L*)	This study, 37
M-miniOVA_257_	MVA-TK-miniOVA	MVA with sequence encoding **M**SIINFEKL, inserted in the TK locus and driven by the VACV p7.5 promoter	Referred to as MVA-SIIN in reference 17
M-ESminiOVA_257_	MVA-TK-ESminiOVA	MVA with sequences encoding **MRYMILGLLALAAVCSA**ASIINFEKL	This study
M-OVA	MVA-TK-OVA	MVA with the full-length chicken ovalbumin gene inserted in the TK locus and driven by VACV p7.5 promoter	19
M-miniOVA_257_-CPX12 + 203	MVA-TK-miniOVA- A11R/A12L-Cowpox12 + 203	M-CPX12 + 203 with sequence encoding **M**SIINFEKL, inserted in the TK locus and driven by the VACV p7.5 promoter	This study
M-OVA-CPX12 + 203	MVA-TK-fullOVA- A11R/A12L-Cowpox12 + 203	M-CPX12 + 203 MVA with the full-length chicken ovalbumin gene inserted in the TK locus and driven by VACV p7.5 promoter	This study

a Name used in this paper.

b Full name refers to the parental virus and the loci of insertion, followed by the inserted gene.

c Boldface indicates the signal sequence from adenovirus E3/19, inserted in the TK locus and driven by the VACV p7.5 promoter.

Unmodified modified vaccinia Ankara (MVA) was originally a gift from B. Moss (National Institutes of Health, Bethesda, MD). MVA stocks were grown and titrated on BHK-21 cells using standard techniques. Cowpox virus, strain Brighton Red, was a gift from G. L. Smith (University of Cambridge, UK). All virus stocks were purified by ultracentrifugation through a 36% sucrose cushion and titers determined by immunostaining on BHK-21 cells.

### Generation of recombinant viruses.

Sequences of CPXV12 and CPXV203 and their promoter regions were defined as nucleotide (nt) 13009 to 13355 (complementary) and nt 188519 to 189369, respectively, of GenBank accession no. AF482758. These were cloned by PCR from crude preparations of genomic DNA from a stock of CPXV strain BR into p7.5ins (37). This plasmid allows the insertion of genes into the intergenic space between VACV genes A11R and A12L. Recombinant viruses were generated using homologous recombination between transfer plasmid (based on p7.5ins or pSC11) with genes of interest (CPXV genes, OVA, miniOVA_257_, or ES-miniOVA_257_) and viral genomes, followed by blasticidin/green fluorescent protein (GFP) or 5-bromo-4-chloro-3-indolyl-β-d-galactopyranoside (X-Gal) selection as previously described (37). Briefly, BHK-21 monolayers were infected with MVA at a multiplicity of infection (MOI) of 0.05 PFU/cell in DMEM supplemented with 2% FBS for 1 h at 37°C and 5% CO_2_. Viral inoculum was replaced with a preincubated transfection mix of Lipofectamine 2000 (Invitrogen), transfer plasmid, and DMEM and incubated at 37°C and 5% CO_2_ for 2 h. Transfection mix was replaced with DMEM supplemented with 10% FBS and transfection allowed to proceed for 2 days at 37°C and 5% CO_2_. Virus was then released by 3 freeze-thaw cycles and sonication before isolation. Recombinant viruses were isolated by serial step purification and addition of blasticidin (15 μg/ml) for 2 days. Remaining foci that were GFP^+^ were collected and grown before PCR screening for insert and further plaque purification. For incorporation of Ova or mini-Ova, X-Gal staining was used in conjunction with GFP expression, as the insertion sequence also contained a *lacZ* gene. Final recombinant viruses were purified using sucrose cushion ultracentrifugation, and DNA sequencing was used to confirm correct sequence insertion.

### Infection of mice.

Mice were inoculated with 1.0 × 10^6^ PFU of virus, diluted in phosphate-buffered saline (PBS), by i.p, i.d., or i.v. injection. For i.d. infections, mice were injected in the left ear pinna with 10 μl of virus suspension as previously described (41, 58, 59). Mice were euthanized 7 days after inoculation, and spleens were collected for analyzing the CD8^+^ T cell response.

### Immunization of mice with infected cells to assess cross presentation *in vivo*.

BHK-21 cells were infected with the virus indicated for 7 h with agitation. Cells were collected, washed three times, and resuspended in PBS. Cells were incubated at 60°C for 1 h to inactivate residual inoculum. Cells were counted and each mouse was inoculated with 1.0 × 10^7^ cells via i.p. injection.

### Measuring CD8^+^ T cell responses and flow cytometry.

Methods to measure epitope-specific and overall CD8^+^ T cells in mice infected with virus or immunized with infected cells were as published previously. Briefly, for epitope-specific responses, 1.0 × 10^6^ splenocytes were cultured with 0.1 μM peptide in DMEM (Table 2). After 1 h of stimulation, brefeldin A was added to a concentration of 50 μg/ml before a further 3 h of culture. Cells were then labeled for CD8 (anti-mouse CD8α-phycoerythrin; clone 53.67; BioLegend) diluted in PBS with 2% FBS and intracellular IFN-γ (anti-mouse IFN-γ-allophycocyanin; clone XMG1.2; BioLegend) diluted in PBS with 2% FBS and 0.25% saponin. Flow cytometry was used to identify the fraction of CD8^+^ events that were also IFN-γ^+^. A no peptide control was used for all experiments, and that value was deducted as the background from all samples from that mouse. For the total size of the antiviral CD8^+^ T cell response, splenocytes were stained directly *ex vivo* for surface CD8 (clone 53.67; BioLegend), CD62L (clone MEL-14), and intracellular GzmB (clone GB11). CD8^+^ events that were CD62L^−^ and GzmB^+^ were considered activated by virus infection. Data for all flow cytometry was acquired using an LSR-II flow cytometer (BD Biosciences) and analyzed with FlowJo 8.8.4 software (TreeStar, Ashland, OR).

**TABLE 2:** Peptide epitopes used throughout this study

Name^*a*^	Origin^*b*^	Sequence	MHC-I restriction	Temporal expression^*c*^	Reference
A8_189_	VACV A8R 189-196	ITYRFYLI	H-2Kb	E1.1	68
A8_70_	VACV A8R 70-77	IHYLFRCV	H-2Kb	E1.1	68
A3_191_	VACV A3L 191-199	YSPSNHHIL	H-2Db	I	68
A3_270_	VACV A3L 270-277	KSYNYMLL	H-2Kb	I	68
A19_47_	VACV A19L 47-55	VSLDYINTM	H-2Kb	I	56
A2329_7_	VACV A23L 297-305	IGMFNLTFI	H-2Db	E1.2	68
A42_88_	VACV A42R 88-96	YAPVSPIVI	H-2Db	I	56
A4715_7_	VACV A47L 157-166	YAHINALEYI	H-2Db	E1.2	44
B8_20_	VACV B8R 20-27	TSYKFESV	H-2Kb	E1.1	56
B2_54_	VACV B2R 54-62	YSQVNKRYI	H-2Db	E1.2	68
G8_34_	VACV G8R 34-41	LMYIFAAL	H-2Kb	I	68
J3_289_	VACV J3R 289-296	SIFRFLNI	H-2Kb	E1.2	68
K3_6_	VACV K3L 6-15	YSLPNAGDVI	H-2Db	E1.1	56
L2_53_	VACV L2R 53-61	LNFRFENV	H-2Kb	E1.1	68
N2_60_	VACV N2L 60-68	FLMMNKDEL	H-2Db	E1.1	68
OVA_257_^*d*^	Chicken, Ovalbumin 257-264	SIINFEKL	H-2Kb	69

a Name as referred to throughout this study, denoting the antigen of origin and initial amino acid number.

b Organism and gene of interest as well as the amino acid numbers.

c Kinetic class for VACV genes: E1.1 and E1.2, early; I, intermediate (as described in references 70 and 71).

d Synthesized by Mimotopes. All other peptides were synthesized by GenScript.

### Detection of MHC-I or H-2K^b^-SIINFEKL on infected cells.

At the times shown after infection, cells were labeled with either phycoerythrin-labeled anti-mouse H-2K^b^ (clone AF6-88.5; BioLegend) or Alexa Fluor 647-labeled anti-H-2K^b^/SIINFEKL (clone 25-D1.16; BioLegend). After washing, data were acquired from the cells by flow cytometry as described above.

### *In vitro* activation of OT-I T cells.

For detecting the presence of low quantities of MHC-I/SIINFEKL complexes on cell surfaces, an OT-I T cell activation assay was used. Spleens were collected from naive OT-I mouse, mashed through a 70-μm cell strainer, and subjected to red blood cell lysis. Splenocytes were either pulsed with SIINFEKL peptide at 0.1 μM or remained unpulsed. Pulsed and unpulsed OT-I T cells were washed and mixed before being grown in DMEM supplemented with 10% FBS, 2 mM l-glutamine, 0.1% β-mercaptoethanol, and IL-2 (R&D Systems) for 4 days, with medium renewal including increasing concentrations of IL-2 on days 2 and 3. Lymphocytes were purified by Ficoll-Paque purification and washed prior to use. OT-I T cells were incubated with antigen-presenting cells (at a presenter/effector ratio of 1:5) or with SIINFEKL peptide at 0.1 μM. After 1 h, brefeldin A was added to prevent IFN-γ release, and samples were incubated for a further 3 h. Cells were labeled with anti-CD8 and anti-IFN-γ antibodies and flow cytometry conducted as described above.

### Infection of cells and detection of MHC-I–peptide complexes by multiple reaction monitoring (MRM) mass spectrometry.

DC2.4 cells were counted and 3.0 × 10^8^ cells were infected with VACV at an MOI of 3 PFU/cell for 30 min with shaking. Inoculum was then replaced with DMEM supplemented with 10% FBS, and infection was either stopped by placing on ice or incubated at 37°C and 5% CO_2_ for a further 3 or 6 h. Cells were then collected and washed and virus inactivated by resuspending in PBS with 1 μg/ml 4,5,8-trimethylpsoralen (psoralen; Sigma) and UV irradiation (365 nm, 2 × 15 W; Vilber Lourmat) for 20 min with mixing every 5 min (17). Cells were then washed with PBS to remove psoralen and cell pellets were snap-frozen. Peptide-MHC complexes were immunoprecipitated and peptides eluted and separated as described previously (60, 61). Briefly, cells were lysed in 5 ml of lysis buffer containing 50 mM Tris, pH 8.0, 150 mM NaCl, 0.5% IGEPAL, and cOmplete protease inhibitor cocktail (Roche, Sigma-Aldrich). After clearing the lysate by centrifugation, the peptide-MHC within the supernatant was isolated via immunoaffinity purification using sequential anti-K^b^ (clone Y-3) and anti-D^b^ (clone 28-14-8S) antibodies cross-linked to protein A Sepharose. The bound complexes were dissociated and eluted using 10%, vol/vol, acetic acid, with peptides then fractionated via a C_18_ Chromolith speed rod (5 μm, 50 by 4.6 mm inner diameter; Merck) on an ÄKTAmicro high-performance liquid chromatography (HPLC) system (GE Healthcare) across an increasing gradient of buffer B (80%, vol/vol, acetonitrile, 0.1%, vol/vol, trifluoroacetic acid in water) at a constant flow rate of 1 ml/min. Peptide-containing fractions were then vacuum concentrated (Labconco Centrivap) and resuspended in 20 μl of 0.1%, vol/vol, formic acid in water.

For peptide detection and quantitation, LC-MRM was employed for a panel of known VACV epitopes whose MRM transition parameters have previously been optimized (62, 63). LC-MRM was carried out on a QTRAP 5500 (SCIEX) attached to an Eksigent (SCIEX) nanoLC-Ultra 2D with a cHiPLC-nanoflex system (trap column, 200 μm by 0.5 mm, ChromXP C_18_-CL, 3 μm, 120 Å; analytical column, 75 μm by 15 cm, ChromXP C_18_-CL, 3 μm, 120 Å). Ten microliters of each sample was loaded at a flow rate of 5 ml/min in mass spectrometry buffer A (0.1%, vol/vol, formic acid in water), and samples were separated at 300 nl/min across an increasing gradient of mass spectrometry buffer B (80%, vol/vol, acetonitrile, 0.1%, vol/vol, formic acid in water). Instrument dwell time was set to 10 ms per transition, and MRMs were acquired at unit resolution. An enhanced product ion scan was set to trigger on any MRM transition exceeding 1,000 cps. To approximate peptide quantitation, a separate run of a known quantity of a synthetic version of each peptide was analyzed, and summed peak areas were compared to those obtained in each sample after adjusting for sample loading.

### Statistical analysis.

Statistical analysis was conducted with GraphPad Prism software or with R (64). For comparisons of fewer than two groups, an unpaired two-tailed test was used. For comparisons for experiments with more than two groups, statistical significance was determined by analyzing responses to peptides separately and fitting a linear model with the lmer and lme4 packages (65, 66) and analysis of variance (ANOVA), assessing the interaction between the magnitude of the CD8^+^ response and virus used. Pairwise comparisons were then conducted with the emmeans package (67) using a Tukey’s *post hoc* test for pairwise differences. Statistical significance was accepted at a *P* value of <0.05.

### Data availability.

MRM data have been deposited on the PeptideAtlas and can be accessed via the following link: ftp://PASS01650:RH6395e@ftp.peptideatlas.org/.
